# Evaluation and comparison of mortality scores in status epilepticus patients

**DOI:** 10.1186/s12883-025-04565-y

**Published:** 2025-12-22

**Authors:** Sema Nur Erdem, Kadriye Ağan, Ipek Midi

**Affiliations:** Department of Neurology, School of Medicine, Marmara University, Istanbul, Turkey

**Keywords:** Refractory status epilepticus, INCNS, APACHE II, SAPS II, SOFA, STESS, EMSE, GCS, MRS, Mortality

## Abstract

**Objective:**

Status epilepticus (SE) is a common neurological emergency associated with significant morbidity and mortality, with approximately one-third of patients demonstrating resistance to first-line treatment. Electroencephalography (EEG) plays a critical role not only in the diagnosis of SE but also in its monitoring and prognostication. This study aims to evaluate the performance of SE-specific severity scoring systems—Status Epilepticus Severity Score (STESS) and Epidemiology-Based Mortality Score in Status Epilepticus (EMSE)—in predicting mortality, in comparison with four widely used systemic severity scores: Acute Physiology and Chronic Health Evaluation II (APACHE II), Simplified Acute Physiology Score II (SAPS II), Sequential Organ Failure Assessment (SOFA), and the Inflammation, Nutrition, Consciousness, Neurologic function, and Systemic condition (INCNS) score. Furthermore, the effects on mortality of the Glasgow Coma Scale (GCS) for assessing level of consciousness, the age-adjusted Charlson Comorbidity Index (ACCI) for evaluating comorbidities, and the pre-SE Modified Rankin Scale (mRS) for measuring pre-existing morbidity will also be investigated.

**Methods:**

A total of 200 status epilepticus (SE) episodes in 188 patients with available EEG data, followed over a four-year period, were included in the study. The sensitivity, specificity, accuracy, and area under the curve (AUC) of each scoring system were calculated and compared.

**Results:**

The SOFA score demonstrated the highest performance in predicting mortality (AUC = 0.81). Systemic severity scores such as SAPS II, SOFA, APACHE II, and INCNS were found to be more effective in predicting mortality than the SE-specific scores STESS and EMSE.

**Conclusion:**

The evaluated scoring systems accounted for approximately 48% of mortality among adult SE patients; however, none were sufficient to predict mortality either alone or in combination. Therefore, there is a need for more specific prognostic scoring systems.

## Introduction

SE is a commonly encountered neurological emergency associated with high rates of morbidity, mortality, and disability, particularly frequent following stroke [1]. In the literature, the annual incidence is reported to be approximately 10–40 cases per 100,000 population, with mortality rates ranging between 16% and 39%, positioning SE as the second most common cause after cerebrovascular diseases (CVD) [1, 2]. Moreover, SE accounts for approximately 10% of all epilepsy-related deaths [3].

Mortality and morbidity in SE are influenced by several factors, including etiology, age, male sex, duration of SE, frequency of hospitalizations, comorbidities, and the presence of impaired consciousness at onset [4, 5]. Notably, etiology is responsible for nearly 80% of SE-related mortality, with higher rates observed particularly in patients with acute symptomatic or progressive neurological conditions. It is largely believed that in such cases, mortality results not directly from SE itself but from the underlying brain injury that precipitates SE [4].

An altered level of consciousness at presentation is a critical prognostic indicator in patients with SE, with declining consciousness levels being closely associated with increased mortality rates [6]. Mortality is further exacerbated by the development of drug resistance; while overall mortality in adult SE patients may reach up to 30%, it can escalate to approximately 39% in cases of refractory SE [7].

In patients treated with anesthetic agents and managed within the intensive care unit (ICU), additional clinical factors such as the presence of critical illness, prolonged mechanical ventilation dependency, sepsis, vasopressor requirements, and multi-organ dysfunction substantially contribute to heightened morbidity and mortality risks. To assess disease severity and guide individualized therapeutic strategies in ICU settings, various general prognostic scoring systems, including APACHE II [8], SAPS II [9, 10], GCS [11], and the SOFA score [12], are employed. Although these scoring systems are not specific for neurology patients, the INCNS score [13] has been shown to perform better in terms of prognosis for neurological patient populations [13]. Nevertheless, since the INCNS score is not SE-specific, two dedicated scoring systems, STESS [14] and EMSE [15], have been proposed to predict outcomes in SE. However, research suggests that these scores are more effective in predicting survival than mortality, characterized by high negative predictive values but relatively low positive predictive values [14–16]. Accurately assessing disease severity and personalizing treatment strategies remains paramount, particularly in critically ill patients with severe systemic comorbidities or refractory SE episodes. Existing prognostic models may be insufficient to fully evaluate such complex clinical scenarios.

Accordingly, the present study aimed to comprehensively assess the demographic characteristics, SE type, semiology, and etiological factors of patients diagnosed with SE; to compare the impact of refractoriness on mortality; and to identify key prognostic factors alongside an evaluation of EEG findings. Moreover, the study sought to compare the prognostic performance of two SE-specific scoring systems (STESS and EMSE) with four general systemic severity scores (APACHE II, SAPS II, SOFA, and INCNS). Finally, the influence of comorbidity burden, calculated using ACCI [17], and pre-SE morbidity, evaluated using mRS [18, 19], on overall prognosis was systematically investigated. In addition, to evaluate whether they provide additional prognostic value, the mSTESS score—combining STESS and mRS—and the EACE score—based on the EMSE but excluding the duration and level of consciousness variables—were also included in the analysis [20].

## Materials and methods

### Study design and patient selection

This single-center, cross-sectional study included all patients retrospectively. Although patient enrollment continued for three months following the ethics committee approval (October 2023), all data were collected and analyzed retrospectively. Patients over 18 years of age, diagnosed and followed up with SE between January 2020 and December 2023, were enrolled. Convulsive status epilepticus (CSE) was defined as ≥ 5 min of continuous seizure activity or ≥ 2 seizures without full recovery of consciousness between events. Non-convulsive status epilepticus (NCSE) was identified according to the Salzburg Consensus Criteria based on electrographic findings in the absence of visible convulsions [21, 22]. The same inclusion and exclusion criteria were applied to all patients. Patients were excluded if they were under 18 years of age, if they were referred to external centers due to lack of available hospital beds, if EEG was not available (required for performing the EMSE score), or if their medical records were incomplete.

EEG recordings were obtained using the standard 10–20 electrode placement system. The first EEG recording lasting at least one hour was evaluated for each patient. EEG patterns were classified according to the EMSE (Epidemiology-based Mortality Score in Status Epilepticus) categories as follows: normal or unspecific EEG, epileptiform discharges, periodic discharges (LPDs or GPDs), ictal patterns (ASIDs), and burst-suppression. All EEGs were independently reviewed by two academic clinical neurophysiologists blinded to each other’s assessments, and any discrepancies were resolved by consensus.

The study was conducted in accordance with the Declaration of Helsinki. The study protocol (No. 09.2023.1423) was reviewed and approved by the Ethics Committee for Medical, Surgical, and Drug Research at the Marmara University School of Medicine on November 3, 2023. The study was approved by the ethics committee, and due to its retrospective nature, the requirement for informed consent was waived. No funding was received for this study.

### Demographic and clinical data

Demographic variables, including age at presentation, sex, chronic comorbidities (recorded to calculate the age-adjusted Charlson Comorbidity Index [ACCI]), and the reasons for hospital or emergency department admission, were collected. Clinical data included the clinical service to which the patient was admitted, total hospital and ICU lengths of stay, the day of hospitalization when SE was detected, and SE duration. Particular emphasis was placed on analyzing the impact of advanced age (> 60 years) on mortality outcomes.

Detailed medical histories were obtained to classify the underlying etiologies. Recorded etiologies included central nervous system (CNS) anomalies, presence or absence of a history of antiseizure drug withdrawal, history of seizures, multiple sclerosis, chronic cerebrovascular disease/brain injury, normal pressure hydrocephalus (NPH), alcohol abuse, drug overdose or medication-related events, head trauma, brain tumors, metabolic disorders, acute cerebrovascular events, acute CNS infections, anoxia (due to prolonged hypoxia or cardiac resuscitation), prior diagnosis of epilepsy or mesial temporal sclerosis, and recent antibiotic use. Antibiotic- and chemotherapy-induced SE cases were grouped under “drug-induced etiologies,” while autoimmune encephalitis was categorized under “CNS infections” due to diagnostic challenges at onset. When patients had pre-existing epilepsy but an acute pathology triggering SE was evident, the acute event was considered the principal etiology. Acute etiologies were defined as those with a time interval of ≤ 7 days between the causative event and SE onset; intervals exceeding 7 days were classified as remote symptomatic etiologies.

Patients whose SE was controlled with the first two lines of therapy without recurrence were classified as non-refractory SE (NRSE). Those requiring general anesthesia to terminate seizures were defined as refractory SE (RSE), and patients exhibiting ongoing clinical or electrographic seizure activity beyond 24 h despite anesthetic agents were categorized as super-refractory SE (SRSE) [23, 24].

The predictive performance of Glasgow Coma Scale (GCS) at presentation, ACCI, pre-SE Modified Rankin Scale (mRS), STESS, mSTESS, EMSE, EACE, APACHE II, SAPS II, SOFA, and INCNS scores for in-hospital mortality were systematically evaluated. Comparisons were made to assess the relative prognostic value of each scoring system.

### Statistical analysis

All statistical analyses were performed using SPSS software version 26.0 (IBM Corp., Armonk, NY, USA). The normality of continuous data was assessed using descriptive statistics, graphical evaluations, and the Kolmogorov-Smirnov test. Categorical variables were expressed as frequencies and percentages, while continuous variables were summarized as means and standard deviations. Comparisons between two groups were conducted using the Mann-Whitney U test. For comparisons among more than two groups, the Kruskal-Wallis H test was used, followed by Dunn’s multiple comparison test for post hoc analyses. Chi-square tests (Pearson’s chi-square and Fisher’s exact test) were applied to compare categorical variables. Logistic regression analyses were used to identify independent predictors of mortality. Receiver Operating Characteristic (ROC) curve analyses were performed to evaluate the predictive accuracy of mortality risk scores, with optimal cutoff values determined using the Youden Index. Diagnostic performances were presented using area under the curve (AUC), positive predictive value (PPV), negative predictive value (NPV), sensitivity, and specificity. Statistical significance was set at *p* < 0.05 with a 95% confidence interval. In addition, a multivariable logistic regression analysis was conducted using the forward stepwise method to identify independent predictors of mortality. Variables with *p* < 0.1 in univariate analyses were included in the model, and the model’s performance was evaluated using the Nagelkerke R² and area under the ROC curve (AUC) values.

As this retrospective study included all consecutive eligible patients (*N* = 188; 87 deaths, 46.3%), no a priori sample-size calculation was performed. Post-hoc power analysis indicated > 80% power (α = 0.05) to detect odds ratios ≥ 1.8 and AUC differences ≥ 0.10. The final logistic-regression model (SOFA, INCNS, ACCI) achieved an events-per-variable ratio of 29:1, exceeding recommended thresholds [25, 26]. 

## Results

### Patient population

**Fig. 1:**
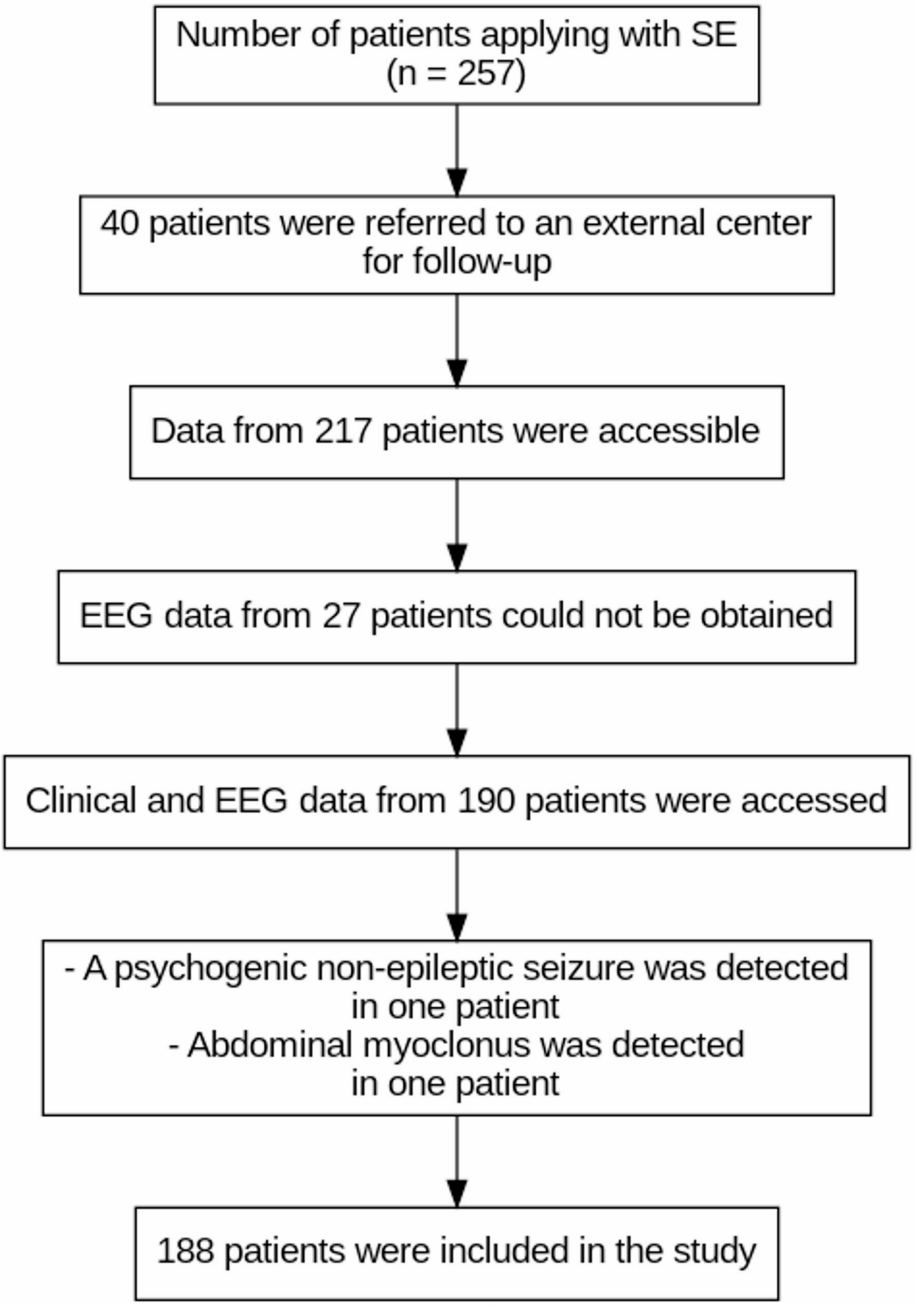
Patients included in the study

Patient data were retrospectively reviewed from archive records and the hospital automation system. Data from patients who had EEG recordings and ongoing follow-up were analyzed. A total of 269 SE (Status Epilepticus) episodes were recorded in 257 patients. Forty patients who were followed up at external centers and 27 patients without EEG recordings were excluded from the study. In addition, two patients — one diagnosed with psychogenic non-epileptic seizures and another with abdominal myoclonus — were also excluded. As a result, a total of 188 patients were included in the study (Fig. 1).

Among the patients, 48.9% (*n* = 92) were over 60 years of age, with the 7th decade being the most common (19.6%, *n* = 37) (Table 1).

**Table 1:** Demographic characteristics and mortality rate of the patients

Variables (*N* = 188)	Category	*n*	%
Age (years)	Mean ± SD	56.99 ± 20.74	
Age group	< 60 years	96	(51.1)
	≥ 60 years	92	(48.9)
Gender	Male	101	(53.7)
	Female	87	(46.3)
Death	Mortality	87	(46.3)

*SD *Standard Deviation

###  Evaluation of mortality with patients’ demographic and clinical data

Mortality was statistically significantly higher among patients aged ≥ 60 years (*p* = 0.001), those admitted to the hospital for surgical reasons (*p* < 0.001), individuals with a known history of neurological disease (*p* = 0.035), those without a history of epilepsy (*p* = 0.003), and patients whose initial level of consciousness was classified as coma (*p* < 0.001). Furthermore, the duration of ICU stay was significantly longer in patients who experienced mortality (Table 2).

**Table 2:** Mortality rates according to patients’ demographic and some clinical characteristics

Variables	Category	Mortality Yes (*n* = 87)	Mortality No (*n* = 101)	Total (*n* = 188)	*P*-value
Age Group	< 60	33 (34.4%)	63 (65.6%)	96 (51.1%)	0.001a*
	≥ 60	54 (58.7%)	38 (41.3%)	92 (48.9%)	
Gender	Male	48 (47.5%)	53 (52.5%)	101 (53.7%)	0.712a
	Female	39 (44.8%)	48 (55.2%)	87 (46.3%)	
Reason for Hospital Admission	Internal Medicine	40 (63.5%)	23 (36.5%)	63 (33.5%)	< 0.001a*
	Surgical	14 (73.7%)	5 (26.3%)	19 (10.1%)	
	Neurological	33 (31.1%)	73 (68.9%)	106 (56.4%)	
Known Neurological Disease	No	31 (58.5%)	22 (41.5%)	53 (28.2%)	0.035a*
	Yes	56 (41.5%)	79 (58.5%)	135 (71.8%)	
History of Epilepsy	No	66 (54.1%)	56 (45.9%)	122 (64.9%)	0.003a*
	Yes	21 (31.8%)	45 (68.2%)	66 (35.1%)	
Initial Consciousness Level	Awake	1 (10.0%)	9 (90.0%)	10 (5.3%)	< 0.001a*
	Drowsy	4 (14.3%)	24 (85.7%)	28 (14.9%)	
	Confused	8 (25.8%)	23 (74.2%)	31 (16.5%)	
	Stupor	15 (55.6%)	12 (44.4%)	27 (14.4%)	
	Coma	59 (64.1%)	33 (35.9%)	92 (48.9%)	
Length of Hospital Stay	Mean ± SD	40.51 ± 34.25	37.62 ± 38.80	38.96 ± 36.69	0.321b
Length of ICU Stay	Mean ± SD	31.24 ± 30.61	24.29 ± 29.33	27.51 ± 30.05	0.010b*
History of Status Epilepticus	No	80 (47.1%)	90 (52.9%)	170 (90.4%)	0.509a
	Yes	7 (38.9%)	11 (61.1%)	18 (9.6%)	

*SD *Standard deviation, *ICU * İntensive care unit

a: Pearson chi-square test

b: Kruskal-Wallis test

*p < 0,05

The mortality rate was statistically singificantly higher in myoclonic SE (Table 3).

**Table 3:** SE, mortality rates according to ILEA semiology

Variables	Category	Total *N*:188 (%)	Mortality *N*:87 (%)	*P*-value
ILEA Motor Symptoms	Prominent	122 (64.9%)	51 (41.8%)	0.094ᵃ
	Not Prominent	66 (35.1%)	36 (54.5%)	
SE Type in Patients with Prominent Motor Symptoms	Convulsive SE (CSE)	80 (65.6%)	28 (35.0%)	0.007ᵇ*
	Myoclonic	35 (28.7%)	22 (62.9%)	
	Focal	7 (5.7%)	1 (14.3%)	
SE Type in Patients Without Prominent Motor Symptoms (NCSE)	With Coma	43 (65.2%)	27 (62.8%)	0.066ᵃ
	Without Coma	23 (34.8%)	9 (39.1%)	
Transition from Prominent Motor Symptoms Group to NCSE	Yes	36 (19.1%)	16 (44.4%)	0.806ᵃ
	No	152 (80.9%)	71 (46.7%)	

*CSE* Convulsive SE, ILEA: The International League Against Epilepsy

a: Pearson chi-square test

b: Fisher’s exact chi-square test

* *p* < 0,05

The mortality rate was significantly higher in the acute symptomatic etiology group (*p* = 0.001). Additionally, mortality was significantly higher in the SE groups associated with metabolic disorders (*p* = 0.016) and hypoxia-anoxia (Table 4).

**Table 4:** Patient mortality rates based on the etiology of SE

Variables	Category	Total *N*:188 (%)	Mortality *N*:87 (%)	*P*-value
ILEA–Etiology	Acute symptomatic	103 (54.8%)	61 (59.2%)	0.001ᵃ*
	Remote symptomatic	40 (21.3%)	13 (32.5%)	
	Progressive symptomatic	31 (16.5%)	11 (35.5%)	
	Idiopathic	14 (7.4%)	2 (14.3%)	

Etiology	Category	TotalN:188 (%)	MortalityN:87 (%)	*P*-value
Associated with antiseizure drugs	No	181 (96.3%)	87 (48.1%)	0.016ᵇ*
	Yes	7 (3.7%)	0 (0.0%)	
Chronic Cerebrovascular Disease (CVD)	No	176 (93.6%)	83 (47.2%)	0.389ᵇ
	Yes	12 (6.4%)	4 (33.3%)	
Drug-related	No	174 (92.6%)	77 (44.3%)	0.057ᵇ
	Yes	14 (7.4%)	10 (71.4%)	
Cryptogenic	No	174 (92.6%)	85 (48.9%)	0.013ᵇ*
	Yes	14 (7.4%)	2 (14.3%)	
Brain tumor	No	162 (86.2%)	77 (47.5%)	0.389ᵃ
	Yes	26 (13.8%)	10 (38.5%)	
Metabolic disorder	No	156 (83.0%)	66 (42.3%)	0.016ᵃ*
	Yes	32 (17.0%)	21 (65.6%)	
Acute CVD	No	150 (79.8%)	70 (46.7%)	0.831ᵃ
	Yes	38 (20.2%)	17 (44.7%)	
Autoimmune/CNS infection	No	177 (94.1%)	85 (48.0%)	0.066ᵇ
	Yes	11 (5.9%)	2 (18.2%)	
Hypoxia–Anoxia	No	160 (85.1%)	67 (41.9%)	0.004ᵃ*
	Yes	28 (14.9%)	20 (71.4%)	

*CNS* central nervous system, *CVD *Cerebrovascular disease, ILEA:The International League Against Epilepsy

a: Pearson chi-square test

b: Fisher's exact chi-square test

* *p*<0,05

### Evaluation of prognostic scores for mortality in patients with SE

The mortality rate in the NRSE group was statistically significantly lower compared to the RSE/SRSE group (*p* < 0.001). A statistically significant difference in mortality rates was observed according to all the prognostic risk assessment scores used in the study (*p* < 0.01 and *p* < 0.001). Overall, it was determined that the average prognostic risk assessment scores of patients who experienced mortality were statistically significantly worse and at higher levels (Table 5).

**Table 5:** Mortality rates according to SE refractoriness and the average prognostic risk scores for mortality

Variables	Mortality Yes (*n* = 87) *N*(%)	Mortality No (*n* = 101) *N*(%)	Total (*n* = 188) *N*(%)	*P*-value^a^
SE Resistance				< 0.001*
NRSE	10 (18.9)	43 (81.1)	53 (28.2)	
RSE/SRSE	77 (57.0)	58 (43.0)	135 (54.8)	

Variables	Mean ± SD	Mean ± SD	Mean ± SD	P-value^b^
SE prior to MRS	2.38 ± 1.64	1.68 ± 1.65	2.01 ± 1.68	0.004*
STESS Score	4.15 ± 1.43	3.10 ± 1.57	3.59 ± 1.59	< 0.001*
mSTESS	5.33 ± 1.70	4.00 ± 1.87	4.62 ± 1.91	< 0.001*
EACE Score	80.29 ± 27.53	65.92 ± 30.65	72.57 ± 30.04	0.002*
EMSE Score	130.00 ± 32.23	101.03 ± 38.42	114.44 ± 38.43	< 0.001*
APACHE-2	20.20 ± 4.80	13.74 ± 6.55	16.73 ± 6.63	< 0.001*
SAPS-2	53.54 ± 13.21	34.93 ± 16.49	43.54 ± 17.67	< 0.001*
SOFA	8.63 ± 2.78	5.30 ± 2.46	6.84 ± 3.09	< 0.001*
INCNS Score	18.00 ± 4.32	10.60 ± 6.64	14.03 ± 6.77	< 0.001*
GCS	4.84 ± 2.58	8.11 ± 3.97	6.60 ± 3.76	< 0.001*
ACCI	5.41 ± 3.31	3.27 ± 2.44	4.26 ± 3.06	< 0.001*

*ACCI* Age-adjusted Charlson Comorbidity Index, *APACHE-2* Acute Physiology and Chronic Health Evaluation-2, *EMSE* Epidemiology-Based Mortality Score in Status Epilepticus, *INCNS* Inflammation, Nutrition, Consciousness, Neurological function, and Systemic status, *mRS* Modified Rankin Scale, *SAPS-2* Simplified Acute Physiology Score-2, *SOFA* Sequential Organ Failure Assessment, *STESS* Status Epilepticus Severity Score, * GCS* Glasgow Coma Scale, *EACE* Etiology, Age, Chronic diseases, EEG, *NRSE* Non Refractory SE, *RSE* Refractory SE, *SRSE* Super Refractory SE

a: Pearson chi-square test

b: Mann-Whitney U test

*p < 0.05

According to the ROC analysis, the most appropriate cut-off values for mortality prediction were as follows: SE prior mRS ≥ 2 (AUC = 0.620), mSTESS ≥ 4 (AUC = 0.703), EACE ≥ 75 (AUC = 0.630), EMSE ≥ 131 (AUC = 0.716), APACHE-II ≥ 17 (AUC = 0.783), SAPS-II ≥ 48 (AUC = 0.808), SOFA ≥ 8 (AUC = 0.810), INCNS ≥ 13 (AUC = 0.809), GCS ≤ 7 (AUC = 0.742), and ACCI ≥ 7 (AUC = 0.692).Among these, the SOFA score demonstrated the best diagnostic performance for predicting mortality, with a sensitivity of 64%, specificity of 79%, and overall accuracy of 72%. Overall, systemic severity scores—particularly SOFA, SAPS-II, and INCNS—showed the highest discriminatory performance for in-hospital mortality, each achieving an AUC of approximately 0.80. In contrast, SE-specific scores such as STESS and EMSE yielded lower predictive accuracy, suggesting that systemic physiological derangements rather than seizure-related characteristics are the predominant determinants of poor outcome in SE (Table 6).

**Table 6:** Diagnostic performance of the most appropriate prognostic risk assessment scores for determining mortality

Variable	Cutpoint	AUC	Sensitivity	Specificity	PPV	NPV	Accuracy
SE prior to MRS	≥ 2	0.620	0.655	0.584	0.576	0.663	0.617
STESS score	≥ 3	0.688	0.897	0.386	0.557	0.813	0.633
mSTESS	≥ 4	0.703	0.839	0.475	0.579	0.774	0.644
EACE score	≥ 75	0.630	0.713	0.584	0.596	0.702	0.644
EMSE score	≥ 131	0.716	0.575	0.782	0.694	0.681	0.686
APACHE-2	≥ 17	0.783	0.782	0.663	0.667	0.779	0.718
SAPS-2	≥ 48	0.808	0.713	0.792	0.747	0.743	0.755
SOFA	≥ 8	0.810	0.644	0.792	0.727	0.688	0.723
INCNS score	≥ 13	0.809	0.897	0.594	0.656	0.869	0.734
GCS	≤ 7	0.742	0.851	0.545	0.617	0.809	0.686
ACCI	≥ 7	0.692	0.494	0.792	0.672	0.645	0.654

*AUC *Area Under the Curve, *Sensitivity *True Positive Rate,* Specificity* True Negative Rate, *PPV *Positive Predictive Value, *NPV* Negative Predictive Value, *Accuracy* Overall correctness, *ACCI* Age-adjusted Charlson Comorbidity Index, *APACHE-2* Acute Physiology and Chronic Health Evaluation-2, *EMSE* Epidemiology-Based Mortality Score in Status Epilepticus, *INCNS* Inflammation, Nutrition, Consciousness, Neurological function, and Systemic status, *mRS* Modified Rankin Scale, *SAPS-2* Simplified Acute Physiology Score-2, *SOFA *Sequential Organ Failure Assessment, *STESS* Status Epilepticus Severity Score, *GCS *Glasgow Coma Scale, *EACE* Etiology, Age, Chronic diseases, EEG

*p < 0,001

To identify independent prognostic risk scores associated with mortality, univariate analyses were initially performed. Scoring systems that demonstrated a statistically significant association with mortality at the 5% significance level were included in a logistic regression model using the forward stepwise method. The optimal model was achieved in the third step and was statistically significant (χ² = 85.344, *p* < 0.001), with a Nagelkerke R² of 0.487 and an area under the ROC curve (AUC) of 0.859, indicating good model performance.

In this multivariable logistic regression analysis, SOFA (OR = 1.294, 95% CI = 1.095–1.528, *p* = 0.002), INCNS (OR = 1.151, 95% CI = 1.061–1.249, *p* = 0.001), and ACCI (OR = 1.211, 95% CI = 1.061–1.383, *p* = 0.005) were identified as independent predictors of mortality. Collectively, these scores accounted for approximately 48% of the variance in mortality outcomes, with a sensitivity of 77.2%, specificity of 75.9%, and overall accuracy of 76.6% (Table 7).

**Table 7:** Independent prognostic risk scores associated with mortality

Variables^(model 3)^	B	SE.	OR[%95 CI]	*P*-value
SOFA	0,257	0,085	1,294 [1,095 − 1,528]	0,002*
INCNS score	0,141	0,042	1,151 [1,061 − 1,249]	0,001*
ACCI	0,191	0,068	1,211 [1,061 − 1,383]	0,005*
Constant	−4,821	0,720	0,008 [0,002 − 0,033]	0,008
Logistic regression model summary	Model method = Forward Stepwise			
	Model, χ²=85,344; *p* < 0,001			
	R^2^_N_ = 0,487; AUC = 0,859			
	Sens.=0,772; Spes.=0,759; Accuracy = 0,766			

* OR* Odds Ratio, *CI *Confidence Inteval, *SH* Standard Error, *AUC *Area Under the Curve, *Sensitivity* True Positive Rate, *Specificity* True Negative Rate, Independent variables excluded from the model, * ACCI* Age-Adjusted Charlson Comorbidity Index, *INCNS* Inflammation, Nutrition, Consciousness, Neurological Function, and Systemic Condition, *SOFA *Sequential Organ Failure Assessment

*p < 0,05

These findings indicate that systemic organ dysfunction and comorbidity burden, rather than seizure-specific variables, play the predominant role in determining mortality in patients with SE.

## Discussion

### Evaluation and discussion of patient demographics, selected clinical parameters, and mortality

In our study, no statistically significant association was found between gender and mortality. Moreover, in alignment with the literature—on which many prognostic scoring systems are also based—advanced age, particularly ≥ 60 years, was significantly associated with increased mortality (*p* = 0.001) [6, 15, 27, 28].

Hospital-acquired mortality was higher among patients admitted due to surgical causes, whereas patients admitted for neurological reasons exhibited significantly lower mortality rates (*p* = 0.010). This finding is consistent with the literature, particularly with the study by Sutter et al., which suggests that the worse initial clinical status of patients at the onset of SE may account for the increased mortality observed in surgical admissions [29].

Furthermore, the absence of a prior history of epilepsy was significantly associated with mortality (*p* = 0.003), corroborating findings from previous studies [30–32]. Similarly, in the STESS scoring system, the lack of a prior epilepsy diagnosis has been associated with a poorer prognosis [14, 33]. Regarding recurrent SE, our study did not identify a statistically significant increase in mortality risk, which is consistent with several previous studies. However, although not observed in our cohort, some published reports suggest that index SE episodes may be associated with higher mortality [34, 35].

Although some studies have identified overall hospital length of stay as an independent predictor of mortality, our study did not find a statistically significant relationship in this regard. However, length of stay in the ICU was significantly associated with mortality (*p* = 0.010) [32]. As expected, prolonged ICU stays often necessitate extended periods of intubation, which increases the risk of complications and thereby contributes to higher mortality rates. Additionally, ICU length of stay was notably prolonged among patients with refractory status epilepticus (RSE), a factor known to adversely affect survival outcomes [4, 29, 36].

Initial level of consciousness is a critical parameter in both SE-specific and general ICU mortality scoring systems. In our study, deterioration in consciousness at onset was significantly associated with increased mortality, consistent with findings from prior research [8, 13, 29, 33].

When evaluating the relationship between initial SE seizure type and mortality, no statistically significant difference was observed between the group with prominent motor symptoms and the nonconvulsive status epilepticus (NCSE) group. However, although not reaching statistical significance, the mortality rate in patients with NCSE was relatively high (54.5%). Previous studies and prognostic scoring systems have associated NCSE with poorer outcomes; nonetheless, this was not statistically validated in our study [6, 29, 33]. A possible explanation is that patients presenting with motor symptoms are more likely to receive immediate treatment in the emergency department, followed by outpatient follow-up, and in many cases, EEG monitoring is not deemed necessary. Although the number of patients presenting with motor symptoms may appear low due to the aforementioned factors, patients with severe motor seizures still require ICU management, and mortality remains high in this subgroup. Another notable finding of our study was the significant association between myoclonic SE and mortality (*p* = 0.007). This is consistent with existing literature, where myoclonic SE has been linked to increased mortality, likely due to its underlying etiology [37].

In line with numerous previous studies, SE caused by acute symptomatic etiologies was significantly associated with increased mortality in our study (*p* < 0.001) [29, 32, 38]. This is believed to be due to the underlying brain damage that leads to SE, as also suggested in other studies [4]. Conversely, poor medication adherence has been associated with better prognosis in the literature, and in our study, no hospital mortality was recorded in cases of SE due to non-adherence to anti-seizure medications (*p* = 0.016) [28, 39]. Anoxic etiologies were also associated with poor outcomes, consistent with previous findings [27, 28, 40]. Acute metabolic disturbances, which are known to carry a high risk for mechanical ventilation, have been demonstrated in the literature to significantly contribute to SE-related mortality. In our study, these etiologies were associated with a high mortality rate of 65.6% (*p* = 0.016) [28, 29, 41]. In contrast, patients with cryptogenic SE exhibited a relatively low mortality rate, aligning with favorable survival outcomes (*p* = 0.013). Although mortality rates in cryptogenic SE vary across studies, most findings suggest that cryptogenic etiologies do not significantly contribute to increased mortality [28, 42].

Similar to other studies, our findings showed that the non-refractory SE (NRSE) group had significantly lower mortality compared to the refractory SE (RSE) and super-refractory SE (SRSE) groups (*p* < 0.001) [6, 27]. However, no significant difference in mortality was found between the RSE and SRSE groups. The increased mortality in refractory cases is thought to result from several factors, including prolonged hospitalization, increased need for intubation and anesthetic agents, and exacerbated neuronal injury associated with refractoriness.

### Evaluation and discussion of prognostic scores determined for SE mortality

In our study, as expected, all prognostic risk assessment scores were statistically significant for mortality and were of similar value (*p* < 0.001). The best diagnostic performance in determining the presence of mortality was found to be associated with the SOFA score (sensitivity = 64%; specificity = 79%; accuracy = 72%). It was determined that independent risk score models for predicting the presence of mortality, when used in combination with other tests, were SOFA, INCNS, and ACCI (sensitivity = 77.2%; specificity = 75.9%; accuracy = 76.6%). In the study by Yuan et al. on critically ill patients in the ICU, when comparing two SE prognostic scores (STESS and EMSE-EACE) with four systemic severity scores (APACHE-2, SAPS-2, SOFA, and INCNS), none of them individually had the desired prognostic power. This study is notable for being the only study to use INCNS for SE mortality [43]. In the same study, the best score for predicting mortality in adults with SE was found to be SAPS-2, but when using a cut-off value of ≥ 18 for APACHE, the highest specificity was obtained [43]. In our study, it was found that the INCNS score, which includes neurological examination, showed very similar results to SOFA, with a sensitivity of 90% and a specificity of about 60%. As in our study, many studies have shown that systemic disease scores such as SAPS-2, SOFA, and APACHE-2 have higher AUC values than SE scores like STESS and EMSE in predicting mortality [44–46]. In the study by Kantenen et al., SOFA score was evaluated as an independent risk factor for mortality in adults with SE, but it was also noted that advanced age, SRSE, and dependence in activities of daily living contributed to this condition [47]. Semmlack et al. concluded that disease severity scores (SAPS-2, SOFA, and APACHE-2) were better at predicting mortality than STESS and that an increased APACHE-2 score was an independent predictor [44]. Many similar studies have also found that APACHE-2 predicts mortality in adults with SE, although inconsistent results have been obtained for mortality in many studies [43, 48–51]. Data from these studies indicate that, in predicting mortality in SE, disease severity scores are more determinant than SE severity scales. It is well-known from various studies that comorbidities can affect mortality; however, as in our study, the ACCI index shows low AUC when used alone but provides better results when used in combination with other prognostic scales [31, 52, 53].

The superior performance of systemic severity scores such as SOFA, SAPS-II, and INCNS likely reflects the pathophysiological complexity of SE, in which mortality is driven not only by cerebral factors but also by widespread systemic dysfunction. Prolonged seizures trigger a cascade of autonomic and metabolic disturbances — including respiratory failure, sepsis, hypotension, and multi-organ injury — that are captured more accurately by systemic scores than by SE-specific indices. While SE-specific tools primarily assess seizure type, duration, and etiology, systemic scores encompass hemodynamic instability, renal and hepatic impairment, and inflammatory burden, all of which have direct prognostic implications. Therefore, the better discriminatory performance of systemic scales underscores that systemic complications, rather than seizure burden alone, dominate outcome trajectories in SE.

Despite achieving excellent discrimination (AUC = 0.859), the combined model explained only 48% of mortality variance (Nagelkerke R² = 0.487), indicating that approximately half of the variability in outcomes remains unexplained. This residual variance most likely reflects unmeasured or unstandardized clinical, systemic, and biological factors that are not captured by conventional severity scores. Variations in treatment-related variables, such as delays in therapy initiation, escalation timing, adherence to protocols, and differences in neurocritical care intensity, may substantially influence outcomes [29, 54–56]. Similarly, seizure-related characteristics—including SE duration, total seizure burden, and specific EEG patterns such as burst suppression or periodic discharges—directly affect neuronal injury but were not quantitatively assessed in the present study [57, 58]. Etiologic heterogeneity within broad diagnostic categories, such as central nervous system infections, may also contribute, as conditions like HSV encephalitis, autoimmune encephalitis, and bacterial meningitis have markedly different prognoses [59]. Furthermore, dynamic in-hospital complications—including nosocomial infections, thromboembolic events, arrhythmias, and acute kidney injury—are not reflected in admission-based severity scores. The prognostic impact of frailty and physiological reserve is likewise underrepresented, as indices such as ACCI and mRS do not fully capture nutritional status, sarcopenia, or overall functional reserve. Variability in EEG monitoring duration, multimodal neuro-monitoring, and individualized management strategies may also influence treatment effectiveness but are difficult to assess retrospectively. Finally, interindividual biological factors, including genetic and pharmacogenomic variability in inflammatory and metabolic pathways, likely play a role in outcome heterogeneity [60].

Collectively, these factors explain why the model demonstrated strong discrimination yet moderate explained variance. Clinically, this suggests that while prognostic scores are useful for distinguishing high- and low-risk groups, individual patient outcomes remain partially unpredictable. Prognostic models should therefore be used to complement, not replace, clinical judgment, and future tools should aim to integrate dynamic, biological, and treatment-related parameters to enhance predictive accuracy and completeness.

This study has several limitations. First, its single-center and retrospective design may limit the generalizability of the findings. Second, only patients with available EEG recordings were included, as EEG data were required for EMSE scoring. This criterion may have introduced selection bias by excluding patients without EEG, particularly those with less severe SE or early mortality. Third, patients with incomplete medical records or missing data were excluded, which may have led to the loss of certain clinical details. Additionally, one patient initially diagnosed with viral encephalitis was later confirmed to have autoimmune encephalitis. Because current mortality scoring systems do not include a separate category for autoimmune etiologies, this case was merged with CNS infections, potentially causing minor heterogeneity within the “autoimmune/CNS infections” group. Furthermore, inter-rater reliability for EEG interpretation was not formally quantified, although two experienced neurophysiologists independently evaluated all EEGs in a blinded manner. As this was a retrospective study including all eligible cases, the power assessment was based on events per variable and minimum detectable effect rather than on prospective sample size estimation. Although the total sample size was comparable to previously published SE cohorts, the number of patients in specific subgroups—particularly those with RSE and SRSE—was relatively small, which may have limited the statistical power of subgroup analyses. Despite these limitations, the study provides valuable insights into the prognostic performance of systemic and SE-specific severity scores in adult patients with SE.

## Conclusion

In the mortality assessment of adult patients with status SE, currently available prognostic scoring systems provide only limited predictive accuracy. Mortality in SE is influenced by numerous interacting factors, including etiology, treatment responsiveness, age, comorbidities, complications developing during hospitalization, and the initial severity of illness. In this study, systemic severity scores—particularly SOFA, SAPS-II, and INCNS—outperformed SE-specific scales in predicting in-hospital mortality, underscoring the dominant role of systemic organ dysfunction and comorbidity burden in SE outcomes. Although the combined model explained 48% of mortality variance, the remaining unexplained variability suggests that unmeasured clinical and systemic factors, such as treatment timing, hospital management differences, and biological heterogeneity, substantially influence prognosis. These findings highlight both the limitations of existing scoring systems and the need for the development of more comprehensive, multifactorial prognostic models. Future tools should integrate systemic and neurological parameters—incorporating treatment response, continuous EEG quantification, and biomarker data—to enhance predictive accuracy and support individualized management strategies in SE.

## Data Availability

The datasets used and analyzed during the current study are available from the corresponding author on reasonable request.
